# Association of Buprenorphine-Waivered Physician Supply With Buprenorphine Treatment Use and Prescription Opioid Use in Medicaid Enrollees

**DOI:** 10.1001/jamanetworkopen.2018.2943

**Published:** 2018-09-28

**Authors:** Hefei Wen, Jason M. Hockenberry, Harold A. Pollack

**Affiliations:** 1 Department of Health Management and Policy, University of Kentucky College of Public Health, Lexington; 2 Department of Health Policy and Management, Emory University Rollins School of Public Health, Atlanta, Georgia; 3 National Bureau of Economic Research, Cambridge, Massachusetts; 4 University of Chicago School of Social Service Administration, Chicago, Illinois

## Abstract

**Importance:**

Expanding treatment for opioid addiction has been recognized as an essential component of a comprehensive national response to the opioid epidemic. The Drug Addiction Treatment Act and its amendments attempted to improve access to treatment by involving office-based physicians in the provision of buprenorphine treatment.

**Objectives:**

To estimate the association of availability of buprenorphine-waivered physicians with buprenorphine treatment use and, secondarily, with prescription opioid use among Medicaid enrollees.

**Design, Setting, and Participants:**

This economic evaluation study used state-level panel data analysis to estimate the association between the number of buprenorphine-waivered physicians and the Medicaid-covered buprenorphine prescribing rate and opioid prescribing rate among Medicaid fee-for-service and managed care enrollees throughout the United States between January 1, 2011, and December 31, 2016.

**Main Outcomes and Measures:**

Buprenorphine prescribing rate and opioid prescribing rate, measured as the number of buprenorphine prescriptions and opioid prescriptions covered by Medicaid on a quarterly basis per 1000 enrollees.

**Results:**

The sample included 1059 quarterly observations. Two additional 100-patient-waivered physicians per 1 000 000 residents (approximately a 10% increase) were associated with an increase in the quarterly number of Medicaid-covered buprenorphine prescriptions of 0.46 (95% CI, 0.24-0.67) per 1000 enrollees and a reduction in the quarterly number of opioid prescriptions of 1.01 (95% CI, −1.87 to −0.15) per 1000 enrollees. Furthermore, 5 additional 30-patient-waivered physicians per 1 000 000 residents (approximately a 10% increase) were associated with an increase in the quarterly number of Medicaid-covered buprenorphine prescriptions of 0.37 (95% CI, 0.22-0.52) per 1000 enrollees and a reduction in the quarterly number of opioid prescriptions of 0.96 (95% CI, −1.85 to −0.07) per 1000 enrollees. A 10% increase in the number of buprenorphine-waivered physicians was associated with an approximately 10% increase in the Medicaid-covered buprenorphine prescribing rate and a 1.2% reduction in the opioid prescribing rate.

**Conclusions and Relevance:**

Expanding capacity for buprenorphine treatment holds the potential to improve access to opioid addiction treatment, which may further reduce prescription opioid use and slow the ongoing opioid epidemic in the United States.

## Key Points

**Question:**

Is the availability of buprenorphine-waivered physicians associated with buprenorphine treatment use and prescription opioid use among Medicaid enrollees?

**Findings:**

In this economic evaluation study using the all-capture Medicaid prescription data between 2011 and 2016, a 10% increase in the number of buprenorphine-waivered physicians was associated with an approximately 10% increase in the Medicaid-covered buprenorphine prescribing rate and a 1.2% reduction in the opioid prescribing rate.

**Meaning:**

Expanding capacity for buprenorphine treatment holds the potential to improve access to opioid addiction treatment, which may further reduce prescription opioid use and slow the ongoing opioid epidemic in the United States.

## Teaser

This economic evaluation study uses Medicaid prescription data from 2011 to 2016 to analyze the association of number of physicians permitted to prescribe buprenorphine for opioid addiction treatment with use of buprenorphine treatment and prescription opioid use among Medicaid enrollees.

## Introduction

The number of prescriptions for opioids in the United States quadrupled between 1999 and 2014.^1^ This increase in opioid prescriptions coincided with a dramatic escalation in opioid addiction and opioid overdose deaths and is considered a leading driver behind the nation’s opioid epidemic.^2,3^ A concerted policy effort has been made over the past decade to regulate opioid prescribing practices by strengthening prescription drug monitoring programs (PDMPs), regulating pain management clinics, and rescheduling hydrocodone combination products.^4,5^ However, restricting legal channels of prescription opioids may have the unintended consequence of pushing those already addicted to opioids to seek alternative, often illegal and more dangerous, drugs and sources.^6,7,8^ Thus, policies that complement supply-side restrictions are needed for tackling the underlying addictive behavior and addressing the ongoing epidemic.^9^

With respect to opioid addiction treatment, medications such as methadone, buprenorphine (including buprenorphine-naloxone), levacetylmethadol, and naltrexone have been approved by the US Food and Drug Administration (FDA) and proven effective in managing withdrawal symptoms and reducing the potential for relapse.^10,11,12^ Nonetheless, most people living with opioid addiction and seeking treatment have not received the treatment recommended for their condition because of the lack of system capacity for providing the treatment.^13^

Historically, provision of opioid agonist medications such as methadone and buprenorphine was allowed only in specialty opioid treatment programs. These programs required patients to take daily medications administrated onsite under the direct observation of program staff.^14^ Even though opioid treatment programs were the only legal providers for agonist treatment, the total number of programs remained constantly low.^15^ Moreover, the programs were unevenly distributed across the nation and lacked integration with general medical care.^15,16,17,18^ As a result, only a fraction of people with opioid addiction were willing and able to come to the programs regularly for treatment.^15,16^

The Drug Addiction Treatment Act of 2000 (DATA 2000) expanded the provision channels of buprenorphine treatment from stand-alone opioid treatment programs to general medical care. Under the DATA 2000, office-based physicians who have a board certification in addiction medicine or psychiatry or complete an 8-hour course of buprenorphine prescribing training are qualified to provide buprenorphine treatment through a waiver.^19^

Although the DATA 2000 waiver mechanism made the first legislative attempt to involve office-based physicians in the provision of buprenorphine treatment, it only allowed a buprenorphine-waivered physician to treat up to 30 patients at any given time (hereafter referred to as *30-patient-waivered physicians*). Subsequently, the Office of National Drug Control Policy Reauthorization Act of 2006 amended the DATA 2000 by raising the patient limit from 30 to 100. Allowing qualified physicians to provide buprenorphine treatment for up to 100 patients (hereafter referred to as *100-patient-waivered physicians*) further helped absorb the unmet treatment need of people with opioid addiction.^15,20^ If the expansion of buprenorphine treatment capacity can effectively increase buprenorphine treatment use, it may hold the potential to reduce the prevalence of opioid addiction, which may further help reduce the number of opioid prescriptions being misused.

Using Medicaid prescription and spending data between 2011 and 2016, our study provides some of the first estimates we know of for the population-level associations between the availability of buprenorphine-waivered physicians and increasing buprenorphine treatment use and, secondarily, reducing prescription opioid use by Medicaid enrollees. Medicaid enrollees, including a growing number of low-income adults in Medicaid expansion states, face high risk of prescription opioid addiction and a high rate of unmet treatment need.^21^ Our findings, therefore, are relevant to the nation’s fight against the opioid epidemic.

## Methods

Our study used deidentified data from publicly available sources and was deemed an exempt human research study by the University of Kentucky institutional review board. We followed the recommendations of the Consolidated Health Economic Evaluation Reporting Standards (CHEERS) reporting guideline and the 24-item CHEERS checklist.^22,23^

### Data

The primary data source for this study is the State Drug Utilization Data from the Centers for Medicare & Medicaid Services. In exchange for federal matching funds, states are required to report to the Centers for Medicare & Medicaid Services on the quarterly amount of prescriptions and spending for all outpatient drugs covered by Medicaid.^24^ We excluded Washington, DC, and Rhode Island, as well as 1 or more quarters of observations in Arizona, Illinois, Kansas, Kentucky, Louisiana, Mississippi, New Jersey, New Mexico, and Oregon from the study data because of inconsistency in state data reporting. The study sample includes 1059 quarterly observations.

We used quarterly, state-level Medicaid fee-for-service and managed care data between the first quarter of 2011 and the second quarter of 2016 (2011 is the first year in which state reporting of Medicaid managed care data became mandatory and nearly complete under the Affordable Care Act data collection requirements). The managed care data capture many high-risk, low-income adult enrollees who recently gained Medicaid coverage under the expansion provisions of the Affordable Care Act or the Section 1115 waiver. Using a study period of 2011 onward not only allows for the inclusion of Medicaid managed care prescription data, but also minimizes the influence of some major common shocks before 2011 or after 2016 that may cause disruptions to buprenorphine use and opioid use. For instance, following the 2010 OxyContin reformulation, there was an initial decline in OxyContin misuse and a shift away from OxyContin to other opioids such as heroin, which then leveled off as the individuals who continued to use these substances changed their preferred route of administration or managed to defeat the abuse-deterrent formulations^25^; the publication of national and state-level (ie, Florida and Washington) guidelines in 2009 and 2010 for appropriate opioid prescribing in chronic pain management, as well as the surgeon general’s warning letter about the opioid crisis in 2016, raised the awareness among physicians and patients of the risks associated with prescription opioid use.^26,27,28,29,30^ Moreover, the Centers for Disease Control and Prevention Guideline for Prescribing Opioids for Chronic Pain,^31^ released in 2016, set the stage for a wave of state laws that recommend or require limits on opioid prescribing duration aligned with the guideline. Although our fixed-effects study design accounted for the nationwide common shocks, states may have responded differently to them. As such, we chose our study window to avoid conflating these policies with the effects of buprenorphine-waivered physicians.

### Measures

The first outcome variable of our study, buprenorphine treatment use, was measured by Medicaid prescriptions for, and spending on, buprenorphine treatment on a quarterly basis per 1000 Medicaid enrollees. Each drug product in the State Drug Utilization Data can be identified by a unique 11-digit, 3-segment National Drug Code number. We excluded a few formulations of buprenorphine generally prescribed for pain management rather than for opioid addiction treatment (eg, Buprenex injectable, Butrans transdermal patch, Belbuca buccal film). eAppendix 1 in the Supplement includes detailed information on the identification of buprenorphine.

The second study outcome was prescription opioid use. In parallel with buprenorphine treatment use, prescription opioid use was measured by quarterly opioid prescriptions and spending per 1000 enrollees. We excluded buprenorphine products classified as medications for opioid addiction treatment from our measurement of prescription opioids. We classified opioid products into 2 categories based on the Controlled Substance Act scheduling: Schedule II opioids and Schedule III-V opioids. The Schedule II opioids are generally considered to have a higher addiction and overdose liability.^32^ Effective October 2014, hydrocodone combination products have been rescheduled from Schedule III to Schedule II.^5^ Accordingly, we classified all hydrocodone combination products as Schedule II opioids throughout the entire study period. Within the category of Schedule II opioids, we further created 6 major subcategories of commonly used, highly addictive opioids: oxycodone, hydrocodone, fentanyl, morphine, oxymorphone, and hydromorphone. In addition to the contemporaneous effect, we also estimated 1-, 2-, 3-, and 4-quarter lagged effects of buprenorphine treatment use on prescription opioid use.

The independent variable of interest was the availability of buprenorphine-waivered physicians. We counted the total number of buprenorphine-waivered physicians per 1 000 000 state residents; then we counted the numbers of 100-patient-waivered physicians and 30-patient-waivered physicians separately. eAppendices 1 and 2 in the Supplement contain detailed information on the identification, measurement, and data sources of the study variables; eTable 1 in the Supplement includes summary statistics of all study variables.

### Statistical Analysis

The main analyses estimated the effects of availability of buprenorphine-waivered physicians on buprenorphine treatment use and the effect on prescription opioid use. We estimated linear weighted least-squares regression models with state and quarter 2-way fixed effects to account for unobserved time-invariant state heterogeneity as well as national secular trends and common shock related to the study outcomes^33^ (eg, rising public awareness of the role of opioids in pain management and the role of buprenorphine in opioid addiction treatment and the nationwide leveling off and gradual reduction in the annual opioid prescribing rate^1^). The secondary analyses used a 2-stage least-squares instrumental variable model, which is analogous to a mediation analysis,^34,35^ to provide further evidence that buprenorphine treatment use served as one of the key pathways from expanding the availability of buprenorphine-waivered physicians to reducing prescription opioid use. eAppendix 3 in the Supplement explains the mediation analyses in detail.

All analyses used sample weights (weighted by the state populations) and standard errors were clustered at the state level using Stata/SE statistical software version 15 (StataCorp LLC).^36^ The clustered standard errors allow for arbitrary within-state correlation in error terms but assume independence across the states.^37^ Statistical significance was assessed at *P* < .05 using 2-tailed tests. The main analyses were adjusted for overall physician supply, general economic conditions, and concurrent policies that may be correlated with prescription opioid use and buprenorphine treatment use by Medicaid enrollees (eg, state prescription drug monitoring program adoption and mandates, pain clinic regulations, Medicaid expansions, and medical marijuana policies). Sensitivity analyses also included group-specific linear trends to account for the unobserved groupwide confounding factors such as trajectory pattern and public sentiment that evolve over time at a constant rate. eAppendix 2 in the Supplement includes detailed information on the model specifications of main analyses and sensitivity analyses. eTables 2 to 7 in the Supplement include detailed estimates from main analyses and sensitivity analyses.

## Results

**Figure 1.**
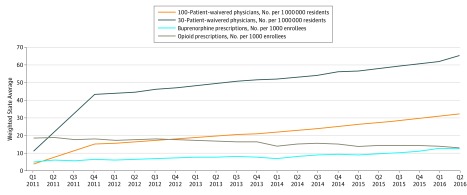
Trends of Buprenorphine-Waived Physicians, Buprenorphine Use, and Prescription Opioid Use Upward trends in the quarterly numbers of 100-patient-waivered physicians and 30-patient-waivered physicians were associated with an upward trend in the quarterly number of Medicaid-covered buprenorphine prescriptions and a downward trend in the quarterly number of opioid prescriptions.

Figure 1 shows upward trends in the quarterly numbers of 100-patient-waivered physicians and 30-patient-waivered physicians, coinciding with an upward trend in the quarterly number of Medicaid-covered buprenorphine prescriptions and a downward trend in the quarterly number of opioid prescriptions.

**Figure 2.**
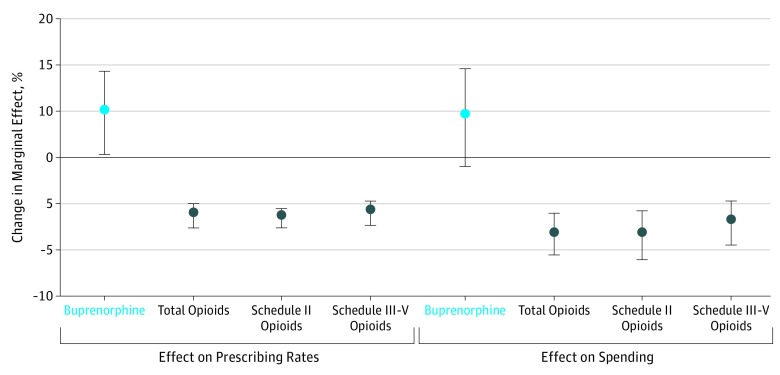
Effect of 10% Increase in Buprenorphine-Waived Physicians on Buprenorphine and Opioid Prescriptions and Spending Dots indicate point estimates of the relative percentage changes; bars indicate 95% confidence intervals. The point estimates and 95% confidence intervals were calculated by combining the estimates from Table 1 and Table 2. The small discrepancy between the figure and the tables was due to the fact that a 10% increase in 100-patient-waivered physicians is 2.085 per 1 000 000, rather than 2 per 1 000 000 (Table 1), and a 10% increase in 30-patient-waivered physicians is 4.886 per 1 000 000, rather than 5 per 1 000 000 (Table 2). The point estimates and 95% CIs in this figure were calculated by multiplying the point estimates and 95% confidence intervals for 100- and 30-patient-waived physicians (eTable 3 in the Supplement) by one-tenth of the means of 100- and 30-patient-waived physicians (eTable 2 in the Supplement), respectively, then adding both products together and dividing it by the means of the outcome variables (eTable 2 in the Supplement). For instance, for buprenorphine prescriptions, we had (0.23 × 2.085 + 0.07 × 4.886) ÷ 8.27 = 9.95%.

Table 1, Table 2, and Figure 2 present the 2-way fixed-effects estimates for the effect of availability of buprenorphine-waivered physicians on buprenorphine treatment use and prescription opioid use by Medicaid enrollees.

**Table 1.** Effect of 2 More 100-Patient-Waivered Physicians per 1 000 000 Residents on Buprenorphine Treatment Use and Prescription Opioid Use at the State, Quarter Level

Outcome Variables per Quarter per 1000 Enrollees^a^	Two-way Fixed-Effect Estimates	
	Marginal Effects (95% CI)^b^	Change, %
Buprenorphine prescriptions, No.	0.46 (0.24 to 0.67)^c^	5.6
All opioid prescriptions, No.	−1.01 (−1.87 to −0.15)^d^	−0.6
Schedule II opioid prescriptions^e^	−0.80 (−1.34 to −0.26)^f^	−0.7
Oxycodone	−0.37 (−0.61 to −0.13)^f^	−1.0
Hydrocodone	−0.34 (−0.64 to −0.04)^d^	−0.5
Oxymorphone	−0.04 (−0.06 to −0.02)^d^	−8.2
Hydromorphone	−0.004 (−0.04 to 0.04)	NA
Morphine	−0.03 (−0.11 to 0.05)	NA
Fentanyl	−0.02 (−0.10 to 0.06)	NA
Schedule III-V opioid prescriptions^e^	−0.21 (−0.45 to 0.02)^g^	−0.5
Buprenorphine spending, $^h^	83.8 (47.8 to 119.6)^c^	4.7
All opioid spending, $^h^	−71.2 (−122.6 to −19.8)^f^	−1.5
Schedule II opioid spending^e^	−68.7 (−127.0 to −10.5)^d^	−1.6
Oxycodone	−24.7 (−43.7 to −5.8)^f^	−1.3
Hydrocodone	−3.78 (−7.70 to 0.14)^g^	−0.4
Oxymorphone	−13.9 (−26.2 to −1.6)^d^	−6.0
Hydromorphone	−5.26 (−13.9 to 3.4)	NA
Morphine	−3.01 (−13.4 to 7.3)	NA
Fentanyl	−18.0 (−41.6 to 5.5)	NA
Schedule III-V opioid spending^e^	−3.86 (−8.96 to 1.24)	NA

Abbreviation: NA, not available.

^a^ Buprenorphine treatment use and prescription opioid use were measured by Medicaid prescriptions for, and spending on, buprenorphine and prescription opioids on a quarterly, per 1000 Medicaid enrollees basis and were population weighted. Buprenorphine products used for opioid addiction treatment were excluded from the measurement of prescription opioids.

^b^ 95% Confidence intervals were calculated based on state-clustered standard errors.

^c^ *P* < .001.

^d^ *P* < .05.

^e^ Opioid products were classified into 2 categories based on the Controlled Substance Act scheduling: Schedule II opioids and Schedule III-V opioids; Schedule II opioids were further classified into 6 major subcategories of commonly used, highly addictive opioids.

^f^ *P* < .01.

^g^ *P* < .10.

^h^ The nominal spending values between 2011 and 2016 were converted to real values based on national monthly Consumer Price Index.

**Table 2.** Effect of 5 More 30-Patient-Waivered Physicians on Buprenorphine Treatment Use and Prescription Opioid Use at the State, Quarter Level

Outcome Variables per Quarter per 1000 Enrollees^a^	Two-way Fixed-Effects Estimates	
	Marginal Effects (95% CI)^b^	Change, %
Buprenorphine prescriptions, No.	0.37 (0.22 to 0.52)^c^	4.5
All opioid prescriptions, No.	−0.96 (−1.85 to −0.07)^d^	**−**0.6
Schedule II opioid prescriptions^e^	−0.84 (−1.40 to −0.25)^f^	−0.7
Oxycodone	−0.55 (−0.93 to −0.17)^f^	−1.4
Hydrocodone	−0.21 (−0.38 to −0.03)^d^	−0.3
Oxymorphone	−0.04 (−0.09 to 0.01)^g^	−8.2
Hydromorphone	−0.004 (−0.10 to 0.09)	NA
Morphine	−0.03 (−0.14 to 0.09)	NA
Fentanyl	−0.003 (−0.08 to 0.07)	NA
Schedule III-V opioid prescriptions^e^	−0.12 (−0.31 to 0.06)	NA
Buprenorphine spending, $^h^	80.5 (25.2 to 135.8)^f^	4.5
All opioid spending, $^h^	−73.0 (−118.7 to −27.2)^f^	−1.6
Schedule II opioid spending^e^	−69.4 (−116.0 to −22.9)^d^	−1.6
Oxycodone	−26.2 (−43.3 to −9.1)^f^	−1.4
Hydrocodone	−5.79 (−10.6 to −0.98)^d^	−0.6
Oxymorphone	−12.8 (−23.4 to −2.23)^d^	−5.6
Hydromorphone	−4.17 (−14.3 to 5.96)	NA
Morphine	−3.03 (−11.1 to 5.05)	NA
Fentanyl	−17.4 (−70.3 to 35.4)	NA
Schedule III-V opioid spending^e^	−3.52 (−7.05 to 0.02)^g^	−0.9

Abbreviation: NA, not available.

^a^ Buprenorphine treatment use and prescription opioid use were measured by Medicaid prescriptions for, and spending on, buprenorphine and prescription opioids on a quarterly, per 1000 Medicaid enrollees basis and were population weighted. Buprenorphine products used for opioid addiction treatment were excluded from the measurement of prescription opioids.

^b^ 95% Confidence intervals were calculated based on state-clustered standard errors.

^c^ *P* < .001.

^d^ *P* < .05.

^e^ Opioid products were classified into 2 categories based on the Controlled Substance Act scheduling: Schedule II opioids and Schedule III-V opioids; Schedule II opioids were further classified into 6 major subcategories of commonly used, highly addictive opioids.

^f^ *P* < .01.

^g^ *P* < .10.

^h^ The nominal spending values between 2011 and 2016 were converted to real values based on national monthly Consumer Price Index (CPI).

We found that 2 additional 100-patient-waivered physicians per 1 000 000 residents (approximately a 10% increase) were associated with an increase in the quarterly number of Medicaid-covered buprenorphine prescriptions of 0.46 (95% CI, 0.24-0.67) per 1000 enrollees, equivalent to a 5.6% increase. Two additional 100-patient-waivered physicians per 1 000 000 residents were further associated with a reduction in the quarterly number of opioid prescriptions of 1.01 (95% CI, −1.87 to −0.15) per 1000 enrollees, a relative 0.6% reduction (Table 1).

We also found that 5 additional 30-patient-waivered physicians per 1 000 000 residents (approximately a 10% increase) were associated with an increase in the quarterly number of Medicaid-covered buprenorphine prescriptions of 0.37 (95% CI, 0.22 to 0.52) per 1000 enrollees or 4.5%, as well as a further reduction in the quarterly number of opioid prescriptions of 0.96 (95% CI, −1.85 to −0.07) per 1000 enrollees, or 0.6% (Table 2).

In addition to the effects on buprenorphine and opioid prescribing rates, expanding the availability of buprenorphine-waivered physicians was also found to be associated with an increase in Medicaid spending on buprenorphine and a reduction in opioid spending. The estimated spending effects on were comparable in size to the prescribing effects (Table 1 and Table 2).

The estimated reductions in prescription opioid use were largely concentrated in Schedule II opioids, especially in oxycodone, hydrocodone, and oxymorphone. These 3 subcategories of Schedule II opioids were commonly prescribed for noncancer pain and have a high potential for addiction and overdose (Table 1 and Table 2). Collectively, these 3 opioids represent two-thirds of all opioid prescriptions and spending covered by Medicaid.

Combining the effects of 100- and 30-patient-waivered physician availability and translating the absolute change into relative percentage change, we estimated that a 10% increase in the number of buprenorphine-waivered physicians (ie, approximately 2 more 100-patient-waivered physicians per 1 000 000 residents and 5 more 30-patient-waivered physicians per 1 000 000 residents based on the 2011-2016 average numbers) was associated with a 10% increase in the Medicaid-covered buprenorphine prescribing rate and a 1.2% reduction in the opioid prescribing rate. In addition to the prescribing effects, a 10% increase in the number of buprenorphine-waivered physicians was also associated with a 9.4% increase in buprenorphine spending and a 3.2% reduction in opioid spending (Figure 2).

**Figure 3.**
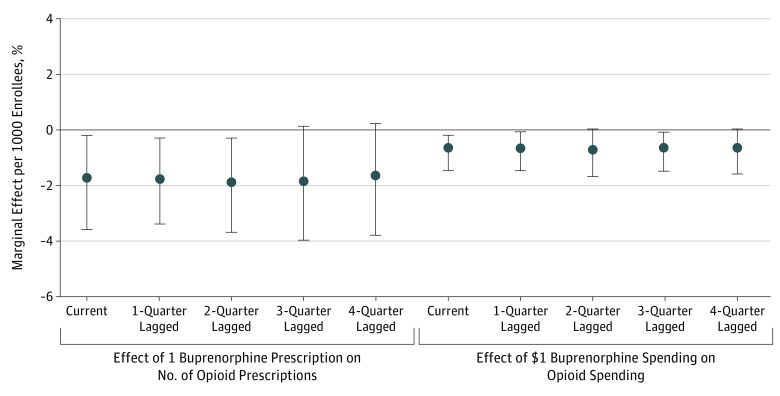
Contemporaneous and Lagged Effect of Buprenorphine Prescribing Rate and Spending on Opioid Prescribing Rate and Spending Dots indicate point estimates of the relative percentage changes; bars indicate 95% confidence intervals.

The mediation analyses provide exploratory evidence that increasing buprenorphine treatment use may have been one of the key pathways from expanding buprenorphine-waivered physician availability to reducing prescription opioid use (Figure 3). We found that an increase in the quarterly number of Medicaid-covered buprenorphine prescriptions by 1 per 1000 enrollees was associated with a reduction in the quarterly number of opioid prescriptions by 1.83 per 1000 enrollees (95% CI, −3.48 to −0.18 per quarter per 1000 enrollees). Translating the absolute change into relative percentage change, a 10% increase in the buprenorphine prescribing rate was associated with a 1.0% reduction in the opioid prescribing rate. Considering our previous fixed-effects estimates that a 10% increase in the number of buprenorphine-waivered physicians was associated with a 10% increase in the buprenorphine prescribing rate and a 1.2% reduction in the opioid prescribing rate, an estimated 83% (ie, 1 ÷ 1.2) of the reduction in opioid prescribing rate associated with expanding buprenorphine-waivered physician availability was through the pathway of increasing the buprenorphine prescribing rate. The mediation analyses for spending effects were consistent with those for prescribing effects. Furthermore, the lagged estimates suggest a significant sustained effect of buprenorphine treatment use on prescription opioid use lasting at least 4 subsequent quarters (Figure 3). The magnitude of the 4-quarter lagged reductions in opioid prescribing rate and spending was over 90% of the magnitude of the contemporaneous reductions.

## Discussion

Our study has direct policy relevance to the ongoing opioid epidemic. On July 6, 2016, the Department of Health and Human Services released a final rule, effective on August 8, 2016, that allows eligible office-based physicians to request approval to treat up to 275 patients. The final rule also includes requirements to ensure that patients treated by these buprenorphine-waivered physicians receive a full array of evidence-based services that minimize the risk of misuse and diversion.^38^ On July 22, 2016, the Comprehensive Addiction and Recovery Act was signed into law, including a provision that further extended the DATA waiver privilege to nurse practitioners and physician assistants.^39^ On March 29, 2017, the Trump administration established a bipartisan Commission on Combating Drug Addiction and the Opioid Crisis and announced new actions to address the epidemic. The commission’s strategy prominently features proposals to improve access to opioid addiction treatment by expanding the capacity for buprenorphine treatment, especially for underserved populations and areas.^40^

An estimated one-third of opioid prescriptions and 40% of opioid spending between 2011 and 2015 were misused, of which Medicaid shared a disproportionately large burden.^41,42,43^ Our findings suggest that expanding capacity for buprenorphine treatment may help address this challenge: the 2-way fixed-effects estimates indicate that a 10% increase in the number of buprenorphine-waivered physicians was associated with a 10% increase in the Medicaid-covered buprenorphine prescribing rate and a 1.2% reduction in the opioid prescribing rate. We also provided exploratory evidence that increasing buprenorphine treatment use may have been 1 of the key pathways from expanding buprenorphine-waivered physician availability to reducing prescription opioid use. The sustained effect of buprenorphine treatment use on prescription opioid use further suggests that buprenorphine treatment may have helped patients to achieve longer-term reduction in opioid use and recovery from opioid addiction, rather than simply acting as a temporary substitute for prescription opioids. As such, the investment in buprenorphine treatment may generate further savings in health care expenditures, criminal justice costs, and labor productivity.

Despite the potential of expanding buprenorphine treatment capacity to reduce prescription opioid use, financial and logistic barriers to buprenorphine treatment still remain. Many states have imposed prior authorization, dosage or duration limits, and extensive counseling documentation requirements on Medicaid coverage for buprenorphine treatment, which may discourage Medicaid patients and physicians from considering such treatment.^44,45^ Furthermore, the uncertain status of the essential health benefits provision of the Affordable Care Act and the lack of explicit inclusion of opioid agonist medication-assisted treatment benefits in the essential health benefits may expose buprenorphine treatment to more stringent future restrictions.^46^

Our study found that 2 additional 100-patient-waivered physicians and 5 additional 30-patient-waivered physician per 1 000 000 residents were associated with an increase in the quarterly number of Medicaid-covered buprenorphine prescriptions by 0.46 (95% CI, 0.24-0.67) and 0.37 (95% CI, 0.22-0.52) per 1000 enrollees, respectively. Given the total population size, Medicaid enrollment, and the guideline-recommended 14- to 30-day refill interval,^19^ our findings imply 6 to 14 additional Medicaid enrollees being prescribed buprenorphine per 100-patient-waivered physician and 2 to 5 additional Medicaid enrollees per 30-patient-waivered physician. To put our estimates into context, existing literature showed that the average caseload of a buprenorphine-waivered physician was about 45% to two-thirds of the approved patient limit.^15,16,47,48^ Considering the overrepresentation of Medicaid enrollees among opioid addiction patients, our Medicaid-focused estimates seem to be consistent with and on the lower side of the existing statistics. One may speculate that some Medicaid enrollees may have faced difficulties in access to buprenorphine-waivered physicians and treatment, or paid out-of-pocket for buprenorphine treatment to circumvent the Medicaid restrictions and requirements.

In light of the remaining financial and logistic barriers and policy uncertainty surrounding access to buprenorphine treatment, active physician participation and a supportive state policy environment are needed to ensure that buprenorphine treatment achieves its full potential in addressing opioid addiction and the ongoing epidemic.^15,47,48,49,50^ Furthermore, future research may investigate whether new buprenorphine-waivered physicians have obtained waivers as a result of a training program requirement or as a recipient of free continuing medical education, as opposed to those who have obtained waivers as a result of specialization in psychiatry and addiction medicine. The new 275-patient-waivered physicians would be another group to examine in future studies.

### Limitations

Our study is subject to the following limitations. First, we only studied opioid prescriptions covered by Medicaid. Furthermore, our aggregate data do not allow us to determine whether and to what extent an estimated change in opioid prescriptions reflects inappropriate use. We expect a significant proportion of the effect of buprenorphine treatment use on prescription opioid use to be on the reduction of misuse, rather than a reduction in appropriate pain management. Nonetheless, our data do not allow us to directly scrutinize this expectation.

Second, although we included only the buprenorphine formulations that are FDA approved for opioid agonist medication-assisted treatment, we cannot rule out the off-label, unapproved use of buprenorphine. Concerns have been voiced over the emerging phenomenon of the misuse of buprenorphine.^51^

Third, our measures of prescription counts did not account for variations in formulation, strength, and dosage. The data lack the information to convert prescription counts to more standardized values such as morphine milligram equivalents. Nonetheless, we provided separate estimates for major subcategories of opioids. The estimates suggest that the reductions in opioid prescriptions were largely concentrated in Schedule II opioids, which, to some extent, helps alleviate the concern about potential substitution of prescriptions with higher morphine milligram equivalents (ie, higher strength and higher dose) for those with lower ones that would otherwise undermine the study findings.

Fourth, as with any observational study, we cannot establish a causal chain from buprenorphine-waivered physician availability, through buprenorphine treatment use, to prescription opioid use. Buprenorphine treatment use constitutes one of the potential pathways from buprenorphine-waivered physician availability to prescription opioid use. There may be other pathways in this causal chain. For instance, once they have received the buprenorphine prescribing training, buprenorphine-waivered physicians may become more aware of the opioid crisis and the problems with overprescribing opioids, thus reducing the use of prescription opioids in pain management. Nonetheless, the consistent results from the main and sensitivity analyses, as well as the supporting evidence from the secondary instrumental variable analyses, strongly suggested that expanding the availability of buprenorphine-waivered physicians has the potential to increase buprenorphine treatment use by Medicaid enrollees and reduce their prescription opioid use.

## Conclusions

Our study uses timely, comprehensive Medicaid administrative data and provides empirical evidence that an increase in the number of physicians qualified to provide buprenorphine treatment was associated with a significant increase in buprenorphine prescriptions and spending covered by Medicaid, which may have induced economically meaningful reductions in Medicaid-covered opioid prescriptions and spending. Our findings suggest that expanding treatment capacity for buprenorphine holds the potential to improve access to opioid addiction treatment, which may further reduce prescription opioid use and help slow the ongoing opioid epidemic in the United States.

## Notes

Supplement. **eAppendix 1.** Identification of Buprenorphine for Medication-Assisted Treatment of Opioid Addiction **eReferences** **eAppendix 2.** Identification & Measurement of Study Variables; Model Specifications of Main Analyses & Sensitivity Analyses **eAppendix 3.** Explanation of Mediation Analysis Using Two-Stage Least Squares-Instrumental Variable (TSLS-IV) Model **eTable 1.** Summary Statistics of the Study Variables at the State, Quarter Level, 2011-2016 **eTable 2.** Summary Statistics of Outcome Variables at the State, Quarter Level for Each Quarter, 2011-2016 **eTable 3.** Effect of Buprenorphine-Waived Physicians on Buprenorphine & Opioid Prescriptions at the State, Quarter Level **eTable 4.** Effect of Buprenorphine-Waived Physicians on Buprenorphine & Opioid Spending at the State, Quarter Level **eTable 5.** Effect of Buprenorphine Prescriptions on Opioid Prescriptions at the State, Quarter Level **eTable 6.** Effect of Buprenorphine Spending on Opioid Spending at the State, Quarter Level **eTable 7.** Sargan–Hansen J Statistics from the Overidentification Test of Instruments
