# Supplementary material for: Association of Buprenorphine-Waivered Physician Supply With Buprenorphine Treatment Use and Prescription Opioid Use in Medicaid Enrollees
Source: JAMA Netw Open. 2018 Sep 28;1(5):e182943. doi: 10.1001/jamanetworkopen.2018.2943 (PMC6324514; doi:10.1001/jamanetworkopen.2018.2943)
Supplement: Supplement. — eAppendix 1. Identification of Buprenorphine for Medication-Assisted Treatment of Opioid Addiction eReferences eAppendix 2. Identification & Measurement of Study Variables; Model Specifications of Main Analyses & Sensitivity Analyses eAppendix 3. Explanation of Mediation Analysis Using Two-Stage Least Squares-Instrumental Variable (TSLS-IV) Model eTable 1. Summary Statistics of the Study Variables at the State, Quarter Level, 2011-2016 eTable 2. Summary Statistics of Outcome Variables at the State, Quarter Level for Each Quarter, 2011-2016 eTable 3. Effect of Buprenorphine-Waived Physicians on Buprenorphine & Opioid Prescriptions at the State, Quarter Level eTable 4. Effect of Buprenorphine-Waived Physicians on Buprenorphine & Opioid Spending at the State, Quarter Level eTable 5. Effect of Buprenorphine Prescriptions on Opioid Prescriptions at the State, Quarter Level eTable 6. Effect of Buprenorphine Spending on Opioid Spending at the State, Quarter Level eTable 7. Sargan–Hansen J Statistics from the Overidentification Test of Instruments [file jamanetwopen-1-e182943-s001.pdf]

Supplementary Online Content

Wen H, Hockenberry JM, Pollack HA. Association of buprenorphine-waivered physician supply with buprenorphine treatment use and prescription opioid use in medicaid enrollees. *JAMA Netw Open*. 2018;1(5):e182943. doi:10.1001/jamanetworkopen.2018.2943

eAppendix 1. Identification of Buprenorphine for Medication-Assisted Treatment of Opioid Addiction
eReferences
eAppendix 2. Identification & Measurement of Study Variables; Model Specifications of Main Analyses & Sensitivity Analyses
eAppendix 3. Explanation of Mediation Analysis Using Two-Stage Least Squares-Instrumental Variable (TSLS-IV) Model
eTable 1. Summary Statistics of the Study Variables at the State, Quarter Level, 2011-2016
eTable 2. Summary Statistics of Outcome Variables at the State, Quarter Level for Each Quarter, 2011-2016
eTable 3. Effect of Buprenorphine-Waived Physicians on Buprenorphine & Opioid Prescriptions at the State, Quarter Level
eTable 4. Effect of Buprenorphine-Waived Physicians on Buprenorphine & Opioid Spending at the State, Quarter Level
eTable 5. Effect of Buprenorphine Prescriptions on Opioid Prescriptions at the State, Quarter Level
eTable 6. Effect of Buprenorphine Spending on Opioid Spending at the State, Quarter Level
eTable 7. Sargan–Hansen J Statistics from the Overidentification Test of Instruments

This supplementary material has been provided by the authors to give readers additional information about their work.

© 2018 Wen H. et al. *JAMA Network Open.*

eAppendix 1. Identification of Buprenorphine for Medication-Assisted Treatment of Opioid Addiction

We used the National Drug Code (NDC) numbers linking to the FDA Orange Book to identify the following sets of Buprenorphine Hydrochloride formulations (including Buprenorphine-Naloxone Hydrochloride formulations) for medication-assisted treatment (MAT) of opioid addiction: (i) Suboxone® sublingual tablets and films, (ii) Subutex® sublingual tablets, (iii) Zubsolv® sublingual tablets, (iv) Bunavail® buccal films, and (v) the generic equivalents of (i), (ii), (iii) and (iv). These medications are FDA-approved for MAT. Any use of them for the treatment of pain or depression is considered an off-label, unapproved use.

We excluded the following sets of Buprenorphine Hydrochloride formulations generally prescribed for pain management: (i) Buprenex® injectable, (ii) Butrans® transdermal patches, (iii) Belbuca® buccal films, and (iv) the generic equivalents of (i), (ii) and (iii). These medications cannot be prescribed for MAT, even by a buprenorphine- waived physician.

We also excluded methadone and naltrexone, the other two FDA-approved medications for MAT, due to their unique funding streams and multiple purposes in treatment: first, methadone and naltrexone are prescribed exclusively in opioid treatment programs and mostly funded by state and local substance abuse treatment agencies, which cannot be fully captured by the study data and may not be directly affected by Medicaid expansions; second, the small segment of the methadone and naltrexone that the study data do capture may not be relevant to opioid addiction treatment, since a considerable proportion of methadone is prescribed for pain management and the majority of naltrexone is for alcohol addiction treatment.

eReferences:

Drug Enforcement Administration (DEA) Office of Diversion Control. Drug and chemical evaluation section: drug use concern-buprenorphine. [Internet]. Washington, DC: U.S. Department of Justice; 2013 Jul. http://www.deadiversion.usdoj.gov/drugs_concern/buprenorphine.pdf

Gordon AJ. The off-label use of sublingual buprenorphine and buprenorphine/naloxone for pain. [Internet]. Providence, RI: PCSS-MAT; 2013 Nov 29. http://pcssmat.org/wp-content/uploads/2014/02/PCSS-MATGuidanceOff-label-bup-for-pain.Gordon.pdf

National Alliance of Advocates for Buprenorphine Treatment (NAABT). Education section FAQ: are there other uses for buprenorphine? [Internet]. Farmington, CT: NAABT; 2015. http://www.naabt.org/faq_answers.cfm?ID=10

U.S. Food and Drug Administration (FDA) Guidance on buprenorphine hydrochloride; naloxone hydrochloride: label. [Internet]. Silver Spring, MD. 2015. http://www.accessdata.fda.gov/drugsatfda_docs/label/2015/207932s000lbl.pdf.

Woodcock J. A difficult balance—pain management, drug safety, and the FDA. *New England Journal of Medicine*. 2009 Nov 26;361(22):2105-7.

Anton RF. Naltrexone for the management of alcohol dependence. *New England Journal of Medicine*. 2008 Aug 14;359(7):715-21.

© 2018 Wen H. et al. *JAMA Network Open.*

eAppendix 2. Identification & Measurement of Study Variables; Model Specifications of Main Analyses & Sensitivity Analyses

Two-Way Fixed Effects Estimation – Main Analysis (Manuscript Tables 1 & 2, Figure 1; Appendix A4 & A5 Columns 1 & 3):

$\textbf{\textit{Buprenorphine Use}}_{s,t} = \alpha_1 + \alpha_2\,\textbf{\textit{\#100-Waived Physician}}_{s,t} + \alpha_3\,\textbf{\textit{\#30-Waived Physician}}_{s,t} + \alpha_4 X_{1\,s,t} + \rho_s + \tau_t + \varepsilon_{s,t}$

$\textbf{\textit{Opioid Use}}_{s,t} = \alpha_1 + \alpha_2\,\textbf{\textit{\#100-Waived Physician}}_{s,t} + \alpha_3\,\textbf{\textit{\#30-Waived Physician}}_{s,t} + \alpha_4 X_{1\,s,t} + \rho_s + \tau_t + \varepsilon_{s,t}$

Two-Way Fixed Effects Estimation – Sensitivity Analysis (Appendix A4 & A5 Columns 2 & 4):

$\textbf{\textit{Buprenorphine Use}}_{s,t} = \alpha_1 + \alpha_2\,\textbf{\textit{\#100-Waived Physician}}_{s,t} + \alpha_3\,\textbf{\textit{\#30-Waived Physician}}_{s,t} + \alpha_4 X_{1\,s,t} + \rho_s + \tau_t + \boldsymbol{\rho_{s}\times t} + \varepsilon_{s,t}$

$\textbf{\textit{Opioid Use}}_{s,t} = \alpha_1 + \alpha_2\,\textbf{\textit{\#100-Waived Physician}}_{s,t} + \alpha_3\,\textbf{\textit{\#30-Waived Physician}}_{s,t} + \alpha_4 X_{1\,s,t} + \rho_s + \tau_t + \boldsymbol{\rho_{s}\times t} + \varepsilon_{s,t}$

Two-Stage Least Squares Instrumental Variables (TSLS-IV) Estimation – Main Analysis (Manuscript Figure 2; Appendix A6 & A7 Column 5): TSLS-IV Stage I: $\textbf{\textit{Buprenorphine Use}}_{s,t} = \alpha_1 + \alpha_2\,\textbf{\textit{\#100-Waived Physician}}_{s,t} + \alpha_3\,\textbf{\textit{\#30-Waived Physician}}_{s,t} + \alpha_4 X_{1\,s,t} + \rho_s + \tau_t + \varepsilon_{s,t}$

TSLS-IV Stage II: $\textbf{\textit{Prescription Opioid Use}}_{s,t} = \beta_1 + \beta_2\,\widehat{\textbf{\textit{Buprenorphine Use}}}_{s,t} + \beta_3 X_{1\,s,t} + \rho_s + \tau_t + \varepsilon_{s,t}$

TSLS-IV Estimation – Sensitivity Analysis I (Appendix A6 & A7 Column 6):

TSLS-IV Stage I: $\textbf{\textit{Buprenorphine Use}}_{s,t} = \alpha_1 + \alpha_2\,\textbf{\textit{\#100-Waived Physician}}_{s,t} + \alpha_3\,\textbf{\textit{\#30-Waived Physician}}_{s,t} + \alpha_4 X_{1\,s,t} + \rho_s + \tau_t + \boldsymbol{\rho_{s}\times t} + \varepsilon_{s,t}$

TSLS-IV Stage II: $\textbf{\textit{Prescription Opioid Use}}_{s,t} = \beta_1 + \beta_2\,\widehat{\textbf{\textit{Buprenorphine Use}}}_{s,t} + \beta_3 X_{1\,s,t} + \rho_s + \tau_t + \boldsymbol{\rho_{s}\times t} + \varepsilon_{s,t}$

TSLS-IV Estimation – Sensitivity Analysis II (Appendix A6 & A7 Column 3):

TSLS-IV Stage I: $\textbf{\textit{Buprenorphine Use}}_{s,t} = \alpha_1 + \alpha_2\,\textbf{\textit{Total \#Buprenorphine-Waived Physician}}_{s,t} + \alpha_3 X_{1\,s,t} + \rho_s + \tau_t + \varepsilon_{s,t}$

TSLS-IV Stage II: $\textbf{\textit{Prescription Opioid Use}}_{s,t} = \beta_1 + \beta_2\,\widehat{\textbf{\textit{Buprenorphine Use}}}_{s,t} + \beta_3 X_{1\,s,t} + \rho_s + \tau_t + \varepsilon_{s,t}$

TSLS-IV Estimation – Sensitivity Analysis III (Appendix A6 & A7 Column 4):

TSLS-IV Stage I: $\textbf{\textit{Buprenorphine Use}}_{s,t} = \alpha_1 + \alpha_2\,\textbf{\textit{Total \#Buprenorphine-Waived Physician}}_{s,t} + \alpha_3 X_{1\,s,t} + \rho_s + \tau_t + \boldsymbol{\rho_{s}\times t} + \varepsilon_{s,t}$

TSLS-IV Stage II: $\textbf{\textit{Prescription Opioid Use}}_{s,t} = \beta_1 + \beta_2\,\widehat{\textbf{\textit{Buprenorphine Use}}}_{s,t} + \beta_3 X_{1\,s,t} + \rho_s + \tau_t + \boldsymbol{\rho_{s}\times t} + \varepsilon_{s,t}$

Ordinary Least Squares (OLS) Estimation – Sensitivity Analysis IV (Appendix A6 & A7 Column 1):

$\textbf{\textit{Prescription Opioid Use}}_{s,t} = \beta_1 + \beta_2\,\textbf{\textit{Buprenorphine Use}}_{s,t} + \beta_3 X_{1\,s,t} + \rho_s + \tau_t + \varepsilon_{s,t}$

OLS Estimation – Sensitivity Analysis V (Appendix A6 & A7 Column 2):

$$\textbf{\textit{Prescription Opioid Use}}_{s,t} = \beta_1 + \beta_2\, \textbf{\textit{Buprenorphine Use}}_{s,t} + \beta_3 X_{1\ s,t} + \rho_s + \tau_t + \boldsymbol{\rho_s \times t} + \varepsilon_{s,t}$$

OLS Estimation vs. TSLS-IV Estimation: In the simple OLS regression, we used the state and quarter fixed effects as well as the group- specific linear trends to isolate the within-state variations in prescription opioid use over time, thereby identifying the effect of buprenorphine use on prescription opioid use. Nonetheless, reverse causality and omitted-variable bias may lead to underestimation in the OLS estimates. First, an increase in prescription opioid use may cause more problems of opioid addiction, thus generating higher potential needs for buprenorphine use. Simply replacing or instrumenting contemporary value of buprenorphine use with its lagged values is problematic if the error terms are correlated over time. This reverse causality from prescription opioid use to buprenorphine use may result in downward-biased OLS estimates. Second, there may be unobserved time-varying factors correlated both with buprenorphine use and with prescription opioid use. Some important unobservables are changes in the composition of state Medicaid enrollees and their underlying risk for addiction, as well as fluctuations in the market price and availability of opioids due to varied intensity of opioid crackdowns and enforcement of prescription regulations. If the omitted variables largely affected both buprenorphine use and prescription opioid use in the same direction, the OLS estimates would be biased towards the null. To address the endogeneity of buprenorphine use with regard to prescription opioid use, we used the number of buprenorphine-waived physicians to isolate the potentially exogenous variation in buprenorphine use. The two instrumental variables in our main analyses are the numbers of 100-patient-waived physicians and 30-patient-waived physicians per 1,000,000 State residents (*100-Waived Physicians,t* & *30-Waived Physician s,t*). In the sensitivity analyses, we used the total number of buprenorphine-waived physicians.

Notes:

s: State, *t*: Year-Quarter;

Buprenorphine Use $_{s,t}$ & *Opioid Use* $_{s,t}$: Medicaid-covered buprenorphine use and prescription opioid use was measured by Medicaid prescriptions for, and spending on, buprenorphine and prescription opioids on a quarterly, per 1,000 Medicaid enrollees basis. The spending data in the CMS State Drug Utilization Data represent pre-rebate amounts reimbursed by Medicaid only, which is a mandatory component of state reporting and consistently reported since the beginning of the Medicaid Drug Rebate Program in 1991. The nominal spending values between 2011 and 2016 were converted to real values based on national monthly Consumer Price Index (CPI);

Data Source: Centers for Medicare and Medicaid Services (CMS). State Drug Utilization Data: https://www.medicaid.gov/medicaid/prescription-drugs/state-drug-utilization-data/index.html;

Buprenorphine Use $_{s,t}$: predicted values derived from the Stage II of the TSLS-IV estimation;

100-Waived Physician $_{s,t}$ & *30-Waived Physician* $_{s,t}$: the counts of the 100-patient-waived physicians and 30-patient-waived physicians per 1,000,000 state residents;

Data Source: Drug Enforcement Agency (DEA). Controlled Substances Act (CSA) Registrants Database;

$X_{1\ s,t}$: a time-varying, state-level vector of overall physician supply, general economic conditions, and concurrent policies that may be correlated with buprenorphine use and prescription opioid use, including:

(i) per capita number of office-based primary care physicians: primary care specialties include general practice, general internal medicine, and family medicine, but exclude pediatrics;
Data Source: Health Resources and Services Administration (HRSA). Area Health Resources Files (AHRF): https://datawarehouse.hrsa.gov/topics/ahrf.aspx;
(ii) per capita number of office-based psychiatrists (including addiction psychiatrists);
Data Source: Health Resources and Services Administration (HRSA). Area Health Resources Files (AHRF): https://datawarehouse.hrsa.gov/topics/ahrf.aspx;
(iii) poverty rate: calculated for the civilian noninstitutionalized population based on household income, household size, and household composition, relative to a set of dollar value thresholds called the “federal poverty level (FPL)”; institutionalized persons, those in military group quarters, and those living in college dormitories, and unrelated children under the age of 15 excluded from the numerator and denominator when calculating the poverty rate;
Data Source: Health Resources and Services Administration (HRSA). Area Health Resources Files (AHRF): https://datawarehouse.hrsa.gov/topics/ahrf.aspx;
(iv) unemployment rate: calculated as the number of unemployed persons divided by the number of persons in the labor force (aged 16 and above); the numerator and denominator excluding the institutionalized persons or those without employment who are not seeking employment;
Data Source: Health Resources and Services Administration (HRSA). Area Health Resources Files (AHRF): https://datawarehouse.hrsa.gov/topics/ahrf.aspx;
(v) “PDMP adoption” indicator: state establishment of Prescription Drug Monitoring Programs;
Data Source: National Alliance for Model State Drug Laws (NAMSDL). Prescription drug monitoring programs: http://www.namsdl.org/prescription-monitoring-programs.cfm;
(vi) “PDMP mandate” indicator: state implementation of mandatory registration and/or mandatory use of the existing PDMPs; Data Source: National Alliance for Model State Drug Laws (NAMSDL). Prescription drug monitoring programs: http://www.namsdl.org/prescription-monitoring-programs.cfm;
(vii) “pain clinic law” indicator: state regulations on pain management clinics,
Data Source: Centers for Disease Control and Prevention (CDC) Public Health Law Program. State laws on prescription drug misuse and abuse: https://www.cdc.gov/phlp/publications/topic/prescription.html
(viii) “ACA Medicaid expansion” indicator: state implementation of Medicaid expansions in compliance with the Medicaid State Plan Amendment (SPA) provision of the ACA;
Data Source: Kaiser Family Foundation (KFF). Status of state action on the Medicaid expansion decision: http://www.kff.org/health-reform/state-indicator/state-activity-around-expanding-medicaid-under-the-affordable-care-act;
(ix) “waiver Medicaid expansion” indicator: state implementation of Medicaid expansions through the Section §1115 waiver; Data Source: Kaiser Family Foundation (KFF). Status of state action on the Medicaid expansion decision: http://www.kff.org/health-reform/state-indicator/state-activity-around-expanding-medicaid-under-the-affordable-care-act;
(x) “early Medicaid expansion” indicator: partial implementation of Medicaid expansions under the “early adoption” provision of the ACA;
Data Source: Sommers BD, Kenney GM, Epstein AM. New evidence on the Affordable Care Act: coverage impacts of early Medicaid expansions.
Health Affairs (Millwood). 2014 Jan 1;33(1):78-87;

(xi) Medicaid managed care penetration rate: measured as the percentage of Medicaid enrollees in comprehensive managed care programs:

Data Source: Centers for Medicare and Medicaid Services (CMS). Medicaid managed care enrollment report; https://www.medicaid.gov/medicaid/managed-care/enrollment/index.html; Kaiser Family Foundation (KFF). Medicaid managed care state-level data; http://www.kff.org/state-category/medicaid-chip/medicaid-managed-care-market-tracker/;

(xii) "medical marijuana law" indicator: state implementation of medical marijuana laws that permit marijuana use for medical purposes; Data Source: Bradford AC, Bradford WD. Medical marijuana laws may be associated with a decline in the number of prescriptions for Medicaid enrollees. *Health Affairs (Millwood)*. 2017 May 1;36(5):945-51;

(xiii) "medical marijuana dispensary" indicator: opening and operation of medical marijuana dispensaries; Data Source: Bradford AC, Bradford WD. Medical marijuana laws may be associated with a decline in the number of prescriptions for Medicaid enrollees. *Health Affairs (Millwood)*. 2017 May 1;36(5):945-51;

ρ_s: State fixed effects, τ_t: Year-Quarter fixed effects; the two-way fixed effects account for the time-invariant state heterogeneity and the national secular trend in buprenorphine utilization that may systematically be correlated with buprenorphine use and prescription opioid use;

$\rho_s \times t$: group-specific linear trends at the Census division level accounting for the unobserved division-wide confounding factors that evolve over time at a constant rate;

All models were population-weighted and were state-clustered to correct for the within-state serial correlation in the error terms;

We excluded D.C. and Rhode Island, as well as one or several quarters of observations in Arizona, Illinois, Kansas, Kentucky, Louisiana, Mississippi, New Jersey, New Mexico, and Oregon, from the study data due to the inconsistency in state data reporting. Regarding the data quality, although the Medicaid SDUD is very timely and useful data, it is prone to unusual patterns in state reporting due to the close linkage to the Medicaid drug rebate program. It is not unusual for states to revise and resubmit data for this system, with retroactive changes sometimes occurring several years after the end of a quarter. Therefore, we have checked with the CMS staff who are responsible for maintenance of the files about the potential data quality concerns and plotted out the state trends in the fee-for-service (FFS) and managed care data separately to check for any unusual, suspicious patterns during the study period. We have also compared the total FFS drug spending against the amounts reported on Form CMS-64, prior to rebates, to see if the orders of magnitude are similar as another quality check. One potential concern, as the reviewer noted, is that prescription drugs paid through Medicaid managed care organizations (MCO) were excluded from the required quarterly data reporting/rebate collection until March 23, 2010, when the ACA expanded the reporting/collection requirement to all the Medicaid MCO carve-ins. By the end of 2011 2nd quarter, the majority of the 22 States using a carve-in approach had collected all the required data and performed data verification checks. A few states revised/resubmitted the complete data later. D.C. is the only state that may still have incompleteness in its first three quarters of 2011 data. Therefore, we also excluded D.C. and started our study period from 2011 onward. Another

potential data quality issue concerns the new adult eligibility group under the ACA Medicaid expansion. At the beginning of 2014, CMS noticed some confusion and inconsistency in state reporting associated with the new adult eligibility group and the increased federal matching rates available to this group. CMS provided states with timely reviews, clarifying information, and technical assistance. The 2014 onward data should have acceptable quality for analysis.

eAppendix 3. Further Explanation of Mediation Analysis Using Two-Stage Least Squares-Instrumental Variable (TSLS-IV) Model

TSLS-IV Stage I: $\textbf{\textit{Buprenorphine Use}}_{s,t} = \alpha_1 + \alpha_2\,\textbf{\textit{\#100-Waived Physician}}_{s,t} + \alpha_3\,\textbf{\textit{\#30-Waived Physician}}_{s,t} + \alpha_4 X_{1\,s,t} + \rho_s + \tau_t + \varepsilon_{s,t}$

TSLS-IV Stage II: $\textbf{\textit{Prescription Opioid Use}}_{s,t} = \beta_1 + \beta_2\,\textbf{\textit{Buprenorphine Use}}_{s,t} + \beta_3 X_{1\,s,t} + \rho_s + \tau_t + \varepsilon_{s,t}$

The two-stage least squares model instrumental variable (TSLS-IV) analysis was used to explored the extent to which buprenorphine treatment use serves as one of the key pathways from expanding the availability of buprenorphine-waived physicians to reducing prescription opioid use. The first stage of the TSLS-IV analysis treated the availability of buprenorphine-waived physicians as the instrument to estimate the variation in buprenorphine treatment use. In the second stage, this quasi-experimental source of variation in buprenorphine treatment use was then treated as the independent variable of interest to estimate the effect on prescription opioid use.

The TSLS-IV estimates can be generalized from the policies aimed at expanding the availability of buprenorphine-waived physicians to a broader range of capacity expansion policies (e.g., allowing nurse practitioners and physician assistants to provide buprenorphine treatment, removing the existing restrictions on Medicaid coverage for buprenorphine treatment).

A key assumption behind the TSLS-IV analysis is that a valid instrument should affect the outcome only through the endogenous independent variable of interest. Conceptually, there may exist other pathways from buprenorphine-waived physician availability to prescription opioid use. For instance, when deciding about waiver application, physicians may consider the overall opioid prescribing rates to gauge the potential treatment needs. However, physicians were unlikely to be able to anticipate the future changes in opioid prescribing rates for Medicaid enrollees in particular, and they were unlikely to decide on apply for the waiver in anticipation of reductions in opioid prescribing rates. Another possible pathway, one may argue, lies in that the required training for buprenorphine-waived physicians may prompt the physicians to become more cautious in opioid prescribing for their pain patients. Nonetheless, the relative small numbers of buprenorphine-waived physicians and pain patients cared by these physicians may not suffice to change the overall patterns of opioid prescribing practices. Furthermore, our TSLS-IV estimates suggest that 83 percent of the reduction in opioid prescribing rate associated with expanding buprenorphine-waived physician availability was through the pathway of increasing buprenorphine prescribing rate. The two instruments allowed for an overidentification test of the exclusion restrictions (Appendix Tables A8 Sargan–Hansen J statistics of the joint overidentification test), which also lends weight to the validity of our instruments.

eTable 1. Summary Statistics of the Study Variables at the State, Quarter Level, 2011-2016

	**Summary Statistics**	
	Mean	*S*
**Outcome Variables** (per quarter per 1,000 enrollees)		
# Buprenorphine Prescriptions	8.27	12.75
# All Opioids Prescriptions	161.64	59.91
~ # Schedule II Opioids Prescriptions	121.98	50.95
~ # Oxycodone Prescriptions	38.18	27.46
~ # Hydrocodone Prescriptions	66.09	33.22
~ # Oxymorphone Prescriptions	0.49	0.90
~ # Hydromoprphone Prescriptions	3.77	3.03
~ # Morphine Prescriptions	6.67	3.87
~ # Fentanyl Prescriptions	3.79	3.22
~ # Schedule III-V Opioids Prescriptions	39.66	12.99
$ Buprenorphine treatment Spending	$1775.	$2122.
$ All Opioid Spending	$4594.	$3449.
~ $ Schedule II Opioid Spending	$4220.	$3302.
~ $ Oxycodone Spending	$1905.	$1510.
~ $ Hydrocodone Spending	$908.5	$633.3
~ $ Oxymorphone Spending	$230.0	$425.7
~ $ Hydromoprphone Spending	$161.2	$529.0
~ $ Morphine Spending	$433.9	$533.1
~ $ Fentanyl Spending	$488.0	$975.1
~ $ Schedule III-V Opioids Spending	$374.4	$230.4
**Key Independent/Instrumental Variables** (per 1,000,000		
# 100-Patient-Limit Waivered Physicians	20.85	14.24
# 30-Patient-Limit Waivered Physicians	48.86	28.54
**Control Variables**		
# Office-Based Primary Care Physicians (per 100,000 residents)	71.87	11.39
# Office-Based Psychiatrists (per 100,000 residents)	8.17	3.35
% Poverty	15.61	2.60
% Unemployment	6.98	1.92
0/1 PDMP Adoption	0.20	0.40
0/1 PDMP Mandate	0.33	0.47
0/1 Pain Clinic Law	0.25	0.43
0/1 ACA Medicaid Expansion	0.23	0.42
0/1 Waiver Medicaid Expansion	0.03	0.17
0/1 Early Medicaid Expansion	0.08	0.27
0/1 Medical Marijuana Law	0.08	0.28
0/1 Medical Marijuana Dispensary	0.06	0.23

eTable 2. Summary Statistics of Outcome Variables at the State, Quarter Level for Each Quarter, 2011-2016

	Summary Statistics	
	Mean	*S.D.*
**Outcome Variables** (per quarter per 1,000		
# Buprenorphine Prescriptions, Year 2011-Year	8.27	12.75
~ Year 2011, Quarter 1	5.24	12.75
~ Year 2011, Quarter 2	6.00	12.75
~ Year 2011, Quarter 3	5.94	12.75
~ Year 2011, Quarter 4	6.47	12.75
~ Year 2012, Quarter 1	6.07	12.75
~ Year 2012, Quarter 2	6.66	12.75
~ Year 2012, Quarter 3	7.09	12.75
~ Year 2012, Quarter 4	7.26	12.75
~ Year 2013, Quarter 1	7.63	12.75
~ Year 2013, Quarter 2	7.88	12.75
~ Year 2013, Quarter 3	8.03	12.75
~ Year 2013, Quarter 4	7.68	12.75
~ Year 2014, Quarter 1	6.86	12.75
~ Year 2014, Quarter 2	8.27	12.75
~ Year 2014, Quarter 3	8.87	12.75
~ Year 2014, Quarter 4	9.38	12.75
~ Year 2015, Quarter 1	8.90	12.75
~ Year 2015, Quarter 2	9.96	12.75
~ Year 2015, Quarter 3	10.46	12.75
~ Year 2015, Quarter 4	10.95	12.75
~ Year 2016, Quarter 1	12.60	12.75
~ Year 2016, Quarter 2	12.78	12.75
# All Opioid Prescriptions, Year 2011-Year	161.64	59.91
~ Year 2011, Quarter 1	185.81	59.91
~ Year 2011, Quarter 2	189.56	59.91
~ Year 2011, Quarter 3	179.03	59.91
~ Year 2011, Quarter 4	182.90	59.91
~ Year 2012, Quarter 1	174.77	59.91
~ Year 2012, Quarter 2	176.00	59.91
~ Year 2012, Quarter 3	183.04	59.91
~ Year 2012, Quarter 4	177.80	59.91
~ Year 2013, Quarter 1	172.17	59.91
~ Year 2013, Quarter 2	169.27	59.91
~ Year 2013, Quarter 3	166.46	59.91
~ Year 2013, Quarter 4	164.77	59.91
~ Year 2014, Quarter 1	141.39	59.91
~ Year 2014, Quarter 2	152.15	59.91
~ Year 2014, Quarter 3	157.97	59.91
~ Year 2014, Quarter 4	152.18	59.91
~ Year 2015, Quarter 1	140.65	59.91

~ Year 2015, Quarter 2	144.02	59.91

~ Year 2015, Quarter 3	145.	59.91
~ Year 2015, Quarter 4	143.	59.91
~ Year 2016, Quarter 1	141.	59.91
~ Year 2016, Quarter 2	130.[illegible]	59.91

eTable 3. Estimated Effect of Buprenorphine-Waived Physicians on Buprenorphine Prescriptions & Opioid Prescriptions at the State, Quarter Level

Effect of 1 More Buprenorphine- Waived Physician per 1,000,000 residents	# **100-Patient-Waived Physicians** (per 1,000,000)						# **30-Patient-Waived Physicians** (per 1,000,000)					
		(1			(2)			(3			(4)	
**Outcome Variables**: (*# Buprenorphine/Opioid prescriptions per quarter per 1,000 enrollees*)												
# Buprenorphine Prescriptions	**0.23****	(0.	*2.8*	**0.32**	(0.	*3.9%*	**0.07****	(0.0	*0.9*	**0.10****	(0.0	*1.0*
# All Opioid Prescriptions	**-**	(0.	-	**-**	(0.	*-0.4%*	**-0.19***	(0.0	-	**-**	(0.0	-
# Schedule II Opioids	**-**	(0.	-	**-**	(0.	*-0.4%*	**-**	(0.0	-	**-**	(0.0	-
~ # Oxycodone	**-**	(0.	-	**-**	(0.	*-0.5%*	**-**	(0.0	-	**-**	(0.0	-
~ # Hydrocodone	**-0.17***	(0.	-	**-**	(0.	*-0.4%*	**-0.04***	(0.0	-	**-**	(0.0	-
~ # Oxymorphone	**-0.02***	(0.	-	**-**	(0.	*-3.8%*	**-0.01**†	(0.0	-	**-**	(0.0	-
~ # Hydromorphone	-	(0.	--	-	(0.	--	-	(0.0	-	-	(0.0	--
~ # Morphine	-0.02	(0.	--	-0.01	(0.	--	-0.01	(0.0	-	-0.01	(0.0	--
~ # Fentanyl	-0.01	(0.	--	-0.01	(0.	--	-	(0.0	-	-	(0.0	--
# Schedule III-V Opioids	**-0.11**†	(0.	-	**-**	(0.	*-0.2%*	-0.03	(0.0	-	-0.02	(0.0	--
# Observations		10			105			10			105	
Group-Specific Linear Trends		N			Yes			N			Yes	

Notes: † *p*<0.10, **p*<0.05, ***p*<0.01, ****p*<0.001;

Marginal effects and significant percentage changes associated with one more buprenorphine-waived physician per 1,000,000 residents; Standard errors in parentheses clustered at the state level;

Shown in Table 1 & Table 2.

eTable 4. Estimated Effect of Buprenorphine-Waived Physicians on Buprenorphine Spending & Opioid Spending at the State, Quarter Level

Effect of 1 More Buprenorphine- Waived Physician per 1,000,000 residents	**# 100-Patient-Waived Physicians** (per 1,000,000)						**# 30-Patient-Waived Physicians** (per 1,000,000)					
		(1			(2)			(3			(4)	
**Outcome Variables**: (*$Buprenorphine/Opioid spending per quarter per 1,000 enrollees*)												
$ Buprenorphine Prescriptions	**41.9****	(9.	*2.4*	**47.6**	(10	*2.7%*	**16.1***	(5.	*0.9*	**19.4**	(4.	*1.1*
$ All Opioid Prescriptions	**-**	(13	-	**-**	(17	*-1.1%*	**-**	(4.	-	**-**	(7.	-
$ Schedule II Opioids	**-33.7***	(15	-	**-**	(16	*-1.1%*	**-**	(4.	-	**-**	(7.	-
~ $ Oxycodone	**-**	(4.	-	**-**	(5.	*-0.8%*	**-**	(1.	-	**-**	(3.	-
~ $ Hydrocodone	**-1.89†**	(1.	-	**-**	(1.	*-0.3%*	**-1.16***	(0.	-	**-**	(1.	-
~ $ Oxymorphone	**-6.94***	(3.	-	**-**	(3.	*-3.8%*	**-2.56***	(1.	-	**-**	(2.	-
~ $ Hydromorphone	-2.63	(2.	--	-3.11	(2.	--	-0.83	(1.	-	-2.06	(1.	--
~ $ Morphine	-1.52	(2.	--	-2.02	(2.	--	-0.61	(0.	-	-1.58	(1.	--
~ $ Fentanyl	-9.02	(6.	--	-14.6	(10	--	-3.49	(5.	-	-4.95	(5.	--
$ Schedule III-V Opioids	-1.93	(1.	--	-3.53	(2.	--	**-0.70†**	(0.	-	**-**	(0.	-
# Observations		10			105			10			105	
Group-Specific Linear Trends		N			Yes			N			Yes	

Notes: † *p*<0.10, **p*<0.05, ***p*<0.01, ****p*<0.001;

Marginal effects associated with one more buprenorphine-waived physician; Standard errors in parentheses clustered at the state level;

Significant percentage changes associated with 10 percent increase in the number of buprenorphine-waived physicians (i.e., approximately two more 100-patient-waived physicians per 1,000,000 residents or five more 30-patient-waived physicians per 1,000,000 residents based on the 2011-2016 average numbers);

Shown in Table 1 & Table 2.

eTable 5. Estimated Effect of Buprenorphine Prescriptions on Opioid Prescriptions at the State, Quarter Level: OLS vs. TSLS-IV Results

Effect of 1 More Buprenorphine		O				TSLS – IV 1				TSLS – IV 2		
	(1)		(2)		(3)		(4)		(5)		(6	
**OUTCOME VARIABLES**: (*# Opioid prescriptions per quarter per 1,000 enrollees*)												
# Schedule II Opioid	**-0.42**†	**(0.25)**	-0.28	(0.17)	**-1.43***	(0.67)	**-1.36***	(0.65)	**-1.46***	(0.72)	**-**	(0.6
~ # Oxycodone	**-0.19***	**(0.08)**	**-**	(0.06)	**-0.82***	(0.33)	**-0.67***	(0.23)	**-**	(0.23)	**-**	(0.2
~ # Hydrocodone	-0.18	(0.12)	-0.12	(0.08)	**-0.55***	(0.27)	**-0.62**†	(0.34)	**-0.67***	(0.31)	**-**	(0.3
~ # Oxymorphone	-0.01	(0.01)	-0.01	(0.01)	**-0.04***	(0.02)	**-0.05**†	(0.03)	**-0.04**†	(0.02)	**-**	(0.0
~ # Hydromorphone	-	(0.02)	-	(0.02)	-0.004	(0.03)	-0.01	(0.03)	-0.003	(0.02)	-	(0.0
~ # Morphine	-0.01	(0.02)	-	(0.02)	-0.02	(0.02)	-0.01	(0.03)	-0.02	(0.02)	-0.01	(0.0
~ # Fentanyl	-0.01	(0.01)	-0.01	(0.02)	-0.01	(0.02)	-0.01	(0.04)	-0.01	(0.01)	-0.01	(0.0
# Schedule III-V Opioid	-0.20	(0.14)	-0.14	(0.10)	-0.34	(0.21)	-0.23	(0.15)	**-0.37**†	(0.24)	-0.27	(0.1
# All Opioid Prescriptions	-0.63†	(0.35)	-0.42	(0.27)	**-1.78***	(0.80)	**-1.59***	(0.78)	**-1.83***	(0.84	**-**	(0.6
~ # One-Quarter Lagged	-0.56	(0.34)	-0.44	(0.30)	**-1.81***	(0.81)	**-1.65**†	(0.85)	**-1.79***	(0.76	**-**	(0.6
~ # Two-Quarter Lagged	-0.65	(0.39)	-0.41	(0.32)	**-1.74***	(0.84)	**-1.56***	(0.73)	**-1.91***	(0.85	**-**	(0.6
~ # Three-Quarter Lagged	-0.64	(0.41)	-0.42	(0.34)	**-1.75***	(0.89)	**-1.58**†	(0.87)	**-1.90**†	(1.04	**-**	(0.8
~ # Four-Quarter Lagged	-0.52	(0.33)	-0.34	(0.25)	**-1.53**†	(0.90)	-1.40	(0.91)	**-1.74**†	(0.97	-1.43	(0.8
**INSTRUMENTS**: (TSLS-IV Stage-I Outcome Variable: *# Buprenorphine Prescriptions per quarter per 1,000 enrollees*)												
# Buprenorphine-Waived Physicians					**0.12** ***	(0.03)	**0.16*****	(0.04)				
~ # 100-Patient-Waived (per	**--**		**--**						**0.23** ***	(0.05)	**0.32* ****	(0.0 5)
~ # 30-Patient-Waived (per	**--**		**--**						**0.08** ***	(0.02)	**0.10* ****	(0.0 2)
F-statistic ‡					26.		21.		28.		33.21	
# Observations	10		1057		10		10		10		10	
Group-Specific Linear Trends	No		Y		No		Yes		No		Y	

Notes: † $p<0.10$, *$p<0.05$, **$p<0.01$, ***$p<0.001$; Standard errors in parentheses are clustered at the state level; ‡ Stock-Yogo weak identification test critical values based on maximal TSLS size of a 5% Wald test of $\beta = \beta_0$: K1=1 & L1=1: 10%: 16.38; 15%: 8.96; 20%: 6.66; 25%: 5.53; K1=1 & L1=2: 10%: 19.93; 15%: 11.59; 20%: 8.75; 25%: 7.25; □ Shown in Figure 3.

eTable 6. Estimated Effect of Buprenorphine Spending on Opioid Spending at the State, Quarter Level: OLS vs. TSLS-IV Results

Effect of $1 More Buprenorphine Spending		O				TSLS – IV 1				TSLS – IV 2		
	(1)		(2)		(3)		(4)		(5)		(6	
**OUTCOME VARIABLES**: (*$Opioid spending per quarter per 1,000 enrollees*)												
$ Schedule II Opioid Spending	**-0.25†**	(0.13)	**-0.27†**	(0.14)	**-0.67****	(0.21)	**-0.73****	(0.25)	**-0.70***	(0.32)	**-**	(0.2
~ $ Oxycodone	**-0.14***	(0.07)	**-0.13†**	(0.07)	**-0.33***	(0.15)	**-0.36***	(0.16)	**-0.37***	(0.17)	**-**	(0.1
~ $ Hydrocodone	**-0.08***	(0.03)	**-0.08***	(0.04)	**-0.08***	(0.04)	**-0.09***	(0.04)	**-0.07†**	(0.04)	**-**	(0.0
~ $ Oxymorphone	-0.01	(0.02)	-0.02	(0.02)	**-0.20†**	(0.12)	**-0.22***	(0.10)	**-0.20***	(0.10)	**-**	(0.1
~ $ Hydromorphone	-	(0.01)	-	(0.01)	-0.02	(0.01)	-0.02	(0.01)	-0.01	(0.01)	-0.02	(0.0
~ $ Morphine	-0.01	(0.02)	-0.01	(0.02)	-0.01	(0.01)	-0.01	(0.01)	-0.01	(0.02)	-0.01	(0.0
~ $ Fentanyl	-0.01	(0.02)	-0.02	(0.02)	-0.01	(0.02)	-0.02	(0.02)	-0.02	(0.03)	-0.02	(0.0
$ Schedule III-V Opioid	-0.07	(0.05)	-0.06	(0.04)	-0.05	(0.03)	**-0.08***	(0.03)	**-0.06†**	(0.03)	**-**	(0.0
$ All Opioid Spending	**-0.32†**	(0.17)	**-0.33***	(0.15)	**-0.71****	(0.23)	**-0.81****	(0.27)	**-0.76***	(0.35	**-0.82****	(0.2
~ $ One-Quarter Lagged	**-0.23†**	(0.13)	-0.31	(0.20)	**-0.72***	(0.35)	**-0.84***	(0.35)	**-0.75***	(0.38	**-**	(0.4
~ $ Two-Quarter Lagged	-0.34	(0.21)	-0.30	(0.21)	**-0.67***	(0.32)	**-0.80†**	(0.42)	**-0.79†**	(0.42	**-**	(0.3
~ $ Three-Quarter Lagged	-0.30	(0.19)	-0.39	(0.26)	**-0.66†**	(0.35)	**-0.74†**	(0.41)	**-0.74***	(0.35	**-**	(0.3
~ $ Four-Quarter Lagged	-0.28	(0.21)	-0.30	(0.22)	**-0.59†**	(0.34)	-0.72	(0.44)	**-0.70†**	(0.38	-0.66	(0.4
**INSTRUMENTS**: (TSLS-IV Stage-I Outcome Variable: *$ Buprenorphine Spending per quarter per 1,000 enrollees*)												
# Buprenorphine-Waived Physicians					**47.2**<br>***	(15.2)	**54.7**<br>***	(13.8)				
~ # 100-Patient-Waived (per	**--**		**--**						**83.7*<br>****	(18.5)	**95.3*****	(21.
~ # 30-Patient-Waived (per	**--**		**--**						**32.2*<br>***	(11.2)	**38.9*****	(9.3
F-statistic ‡					16.		20.		16.		27.05	
# Observations	10		1057		10		10		10		10	
Group-Specific Linear Trends	No		Yes		No		Yes		No		Y	

Notes: † *p*<0.10, **p*<0.05, ***p*<0.01, ****p*<0.001; Standard errors in parentheses are clustered at the state level; ‡ Stock-Yogo weak identification test critical values based on maximal TSLS size of a 5% Wald test of $\beta = \beta_0$: K1=1 & L1=1: 10%: 16.38; 15%: 8.96; 20%: 6.66; 25%: 5.53; K1=1 & L1=2: 10%: 19.93; 15%: 11.59; 20%: 8.75; 25%: 7.25; □ Shown in Figure 3.

eTable 7. Sargan–Hansen J Statistics from the Overidentification Test of Instruments

Sargan–Hansen J Statistics: Overidentification Test of		TSLS – IV 2		
		(		(
**Outcome Variables**:				
# Schedule II Opioid	0.0	(0.94)	0.0	(0.8
~ # Oxycodone	0.0	(0.82)	0.0	(0.8
~ # Hydrocodone	0.0	(0.92)	0.0	(0.9
~ # Oxymorphone	0.0	(0.90)	0.1	(0.7
~ # Hydromorphone	0.3	(0.56)	0.2	(0.6
~ # Morphine	0.8	(0.35)	0.8	(0.3
~ # Fentanyl	0.0	(0.76)	0.0	(0.8
# Schedule III-V Opioid	0.1	(0.70)	0.1	(0.6
# All Opioid Prescriptions	0.0	(0.98)	0.0	(0.9
$ Schedule II Opioid Spending	0.2	(0.64)	0.0	(0.8
~ $ Oxycodone	0.1	(0.66)	0.1	(0.7
~ $ Hydrocodone	0.0	(0.79)	0.0	(0.9
~ $ Oxymorphone	0.1	(0.73)	0.0	(0.9
~ $ Hydromorphone	0.3	(0.58)	0.2	(0.5
~ $ Morphine	0.2	(0.63)	0.0	(0.8
~ $ Fentanyl	1.0	(0.31)	0.4	(0.5
$ Schedule III-V Opioid	0.6	(0.42)	0.3	(0.5
$ All Opioid Spending	0.1	(0.74)	0.0	(0.8
Group-Specific Linear Trends	N		Y	

Notes: *p*-values in parentheses estimated from the joint overidentification test of the numbers of 100- patient-waived physicians and 30-patient-waived physicians per 1,000,000 residents; the Sargan–Hansen J statistics lending statistical support to the exogeneity of both instruments across the board.
